# A New Denoising Method for Belt Conveyor Roller Fault Signals

**DOI:** 10.3390/s24082446

**Published:** 2024-04-11

**Authors:** Xuedi Hao, Jiajin Zhang, Yingzong Gao, Chenze Zhu, Shuo Tang, Pengfei Guo, Wenliang Pei

**Affiliations:** 1 College of Mechanical and Electrical Engineering, China University of Mining and Technology (Beijing), Beijing 100083, China; 18817204610@163.com (J.Z.); gaoyingzong0508@163.com (Y.G.); zhuzhuchz@163.com (C.Z.); 17731679018@163.com (S.T.); 18801077872@126.com (P.G.); 2 CITIC HIC Kaicheng Intelligence, Tangshan 063083, China; peiwenliang@ekaicheng.com

**Keywords:** inspection robots, acoustic signal preprocessing, denoising methods, fault diagnosis

## Abstract

In the process of the intelligent inspection of belt conveyor systems, due to problems such as its long duration, the large number of rollers, and the complex working environment, fault diagnosis by acoustic signals is easily affected by signal coupling interference, which poses a great challenge to selecting denoising methods of signal preprocessing. This paper proposes a novel wavelet threshold denoising algorithm by integrating a new biparameter and trisegment threshold function. Firstly, we elaborate on the mutual influence and optimization process of two adjustment parameters and three wavelet coefficient processing intervals in the BT-WTD (the biparameter and trisegment of wavelet threshold denoising, BT-WTD) denoising model. Subsequently, the advantages of the proposed threshold function are theoretically demonstrated. Finally, the BT-WTD algorithm is applied to denoise the simulation signals and the vibration and acoustic signals collected from the belt conveyor experimental platform. The experimental results indicate that this method’s denoising effectiveness surpasses that of traditional threshold function denoising algorithms, effectively addressing the denoising preprocessing of idler roller fault signals under strong noise backgrounds while preserving useful signal features and avoiding signal distortion problems. This research lays the theoretical foundation for the non-contact intelligent fault diagnosis of future inspection robots based on acoustic signals.

## 1. Introduction

With the rapid development of intelligent coal mining technology, more and more intelligent equipment is being applied in coal mines. Most coal mines transform towards the production method of “reducing manpower by digitalization, minimizing human intervention by automation, and requiring no human presence by intelligence”, leading to significant improvements in coal mine safety and production [1]. There are six main systems in coal mines: mining systems, tunneling systems, electromechanical systems, transportation systems, ventilation systems, and drainage systems. The transportation system stands out as one of the most critical components. Challenges such as equipment reliability, costly repairs after failures, and low levels of predictive maintenance during long-distance transportation in the coal industry have driven the development of intelligent solutions for conveyor systems. Achieving real-time monitoring and fault diagnosis of mine belt conveyors has become one of the research focuses [2]. Currently, more and more inspection robots have been applied to the monitoring operation of belt conveyors, enabling functionalities such as audio–video capture, infrared temperature measurement, gas detection, intrusion object identification, wireless communication, data storage and retrieval, fixed-point monitoring, automatic power monitoring, and autonomous recharging. For the monitoring of coal mine transportation systems, traditional vibration sensors have problems such as large number and difficulty in signal transmission. In intelligent inspection, using sound and visual sensors to achieve predictive maintenance is one of the research focuses.

According to a statistical analysis, 79% of downtime is caused by roller failures. Visual sensors alone cannot effectively identify problems such as roller jamming and abnormal bearings. Therefore, the diagnosis of roller faults based on acoustic signals is of great significance [3]. Due to the long working distances, large number of rollers, and complex underground environments, the collected acoustic signals will be affected by coupling interference such as that from the components of environmental noise and non-periodic transient impact caused by abnormal acoustics. Therefore, in the context of the intelligent inspection of the operational status of idler rollers, a critical challenge is how to reduce signal redundancy, enhance the denoising capabilities during data preprocessing, and improve the diagnostic proficiency of acoustic measurement methods during non-contact inspections.

Recently, numerous scholars have conducted research on denoising algorithms for both acoustic and vibration signals. Huang et al. [4] introduced the Empirical Mode Decomposition (EMD) algorithm, which adaptively decomposes nonlinear and non-stationary signals into multiple components. The Empirical Mode Decomposition method is utilized to decompose noisy signals, and relevant components are selected for superimposed reconstruction. Zhang et al. [5] solved the problem of severe noise contamination in non-stationary signals from flood discharge structures. They proposed a denoising method based on Complete Ensemble Empirical Mode Decomposition with Adaptive Noise (CEEMDAN) and singular value decomposition (SVD), validating the effectiveness of noise filtering through practical examples. Zhang et al. [6] combined Empirical Mode Decomposition with multi-point optimal minimum entropy deconvolution. They reconstructed gear vibration signals by selecting Intrinsic Mode Function (IMF) components with larger kurtosis values, achieving signal denoising for gearboxes. Zhang et al. [7] proposed a novel vibration signal denoising method based on an improved adaptive noise complementation system, Empirical Mode Decomposition, permutation entropy, and singular value decomposition. Experimental validation confirmed its effectiveness for the vibration signals of hydroelectric units. Addressing the weak adaptive capability in the denoising process of partial discharge signals from switchgear equipment, Jin et al. [8] introduced a novel Adaptive Integrated Empirical Mode Decomposition method. This approach adaptively selects Intrinsic Mode Functions for denoising reconstruction, providing a new strategy for preprocessing PD signals in switchgear. In response to the challenge of not being able to choose the most suitable wavelet mother function based on different tasks when using wavelet transform for denoising, Ngui W.K. et al. [9] proposed a novel technology to determine the optimal wavelet mother function through quantitative methods. Hua T. et al. [10] presented a denoising method for laser radar echo signals based on Parameter-Optimized Variational Mode Decomposition (VMD) combined with Hausdorff Distance (HD) and wavelet transform (WT). The simulation and experimental results indicated a higher effectiveness and robustness compared to traditional denoising methods, improving the ranging performance of laser radar in adverse environments. Ali M.N. et al. [11] investigated the impact of wavelet denoising algorithms on heart acoustic signals under different wavelet functions and decomposition levels. Through experiments, they demonstrated that the decomposition level and threshold type are crucial parameters influencing the denoising algorithm. Long J. et al. [12] addressed the limitation of existing denoising algorithms in simultaneously suppressing various types of noise. They proposed a novel approach combining variational mode decomposition and wavelet transform, proving through simulation and experiments that this method effectively denoises while preserving the original signal features. Baldazzi et al. [13] explored a stationary wavelet transform denoising algorithm for electrocardiographic signals. The experimental results showed that this method improves the signal-to-noise ratio of electrocardiographic signals while preserving the original features. Zhang et al. [14] combined adaptive noise Complete Ensemble Empirical Mode Decomposition with wavelet packet decomposition to denoise MC spindle vibration signals, effectively eliminating noise. Xie et al. [15] merged local projection with wavelet packet decomposition. Through multiple iterative denoising using the local projection method combined with wavelet packet decomposition, they successfully suppressed medium- to high-intensity noise. He et al. [16] utilized Empirical Mode Decomposition to process effective Intrinsic Mode Function components of acoustic emission signals generated during welding using wavelet packet decomposition. This approach effectively reduced the noise in acoustic emission signals. He et al. [17] proposed a new threshold considering temporal scale correlation, using the propagation characteristics of wavelet coefficients to determine the threshold. The simulation results indicated that the proposed method has good denoising effects. Yang et al. [18] presented a denoising model that combines a dual-parameter threshold quantization function with wavelet packet algorithms. The experimental validation demonstrated the feasibility and superiority of this method for denoising pipeline valve leakage signals. Li et al. [19] proposed an adaptive wavelet threshold denoising algorithm based on wavelet transform, specifically designed for the noise characteristics of low-altitude flying acoustic target signals. The algorithm achieved multi-scale segmentation through the adaptive adjustment of the threshold function, significantly improving the signal-to-noise ratio and denoising effectiveness. Xu et al. [20] proposed a two-level denoising framework with singular value decomposition and adaptive wavelet denoising to address the problem of weak lidar echo signals. The joint denoising performance of singular value decomposition and adaptive wavelet denoising under Gaussian white noise was simulated and analyzed. Tang et al. [21] addressed the problem of local discharge signals from equipment being susceptible to contamination by on-site white noise. They proposed a method that combines wavelet thresholding and total variation denoising using convex optimization theory. Through experiments, they validated that the proposed algorithm exhibits a superior denoising performance compared to other denoising models. Jang et al. [22] selected the optimal wavelet mother function and decomposition levels based on a quantitative analysis. The feasibility of this denoising method in improving the quality of Doppler electrocardiograms has been verified through experiments.

Numerous scholars have developed new denoising models that have successfully implemented signal preprocessing in various fields. But there has been relatively limited research in the context of acoustic signal denoising for belt conveyor rollers. The most existing methods have insufficient effects on noise reduction and cannot effectively extract fault feature information. In order to reduce the impact of a strong noise environment on the preprocessing of acoustic and vibration signals of belt conveyor rollers during inspection, and to improve the generalization and noise reduction ability of existing noise reduction models, two aspects of research are presented in this paper:

(1) The biparameter and trisegment threshold function (BT) is proposed to address the pseudo-Gibbs problem caused by the mutation of the hard threshold function and soft threshold function. This function can adapt to signals with different characteristics through flexible factor adjustments. The feasibility and advantages of this function are theoretically demonstrated, providing a theoretical foundation for signal denoising in the intelligent diagnosis process of inspection robots.
(2) To verify the denoising characteristics of the new threshold function, comparative experiments are carried out using a controlled variable approach. The experiments maintain a constant threshold, wavelet basis functions, and decomposition levels while only changing the threshold function. Denoising preprocessing is applied to two types of artificially noised simulated signals and experimental signals. A quantitative analysis is performed using three evaluation metrics: the Normalized Cross-Correlation (NCC), Root Mean Square Error (RMSE), and signal-to-noise ratio (SNR). The feasibility and advantages of the proposed threshold function are validated with the experiments.

## 2. Theoretical Research on Denoising Model

### 2.1. The Denoising Model of the BT-WTD Algorithm

#### 2.1.1. Principle of Wavelet Threshold Denoising

The mathematical model of the original one-dimensional signal containing noise can be represented as

(1) $$S_{\left(t_{i}\right)}=f_{\left(t_{i}\right)}+n_{\left(t_{i}\right)}\;i=0,\;1,\;2,\;\ldots ,\;N-1$$

In the formula, $f_{\left(t_{i}\right)}$ is a valid signal; $n_{\left(t_{i}\right)}$ is noise signals.

The core principle of wavelet decomposition is to use the Mallat tower algorithm to reduce the order of the signal. The wavelet decomposition process requires the selection of a wavelet basis function to perform multi-scale wavelet decomposition on the signal to be processed. The decomposition at each scale can be expressed as in Equation (2):

(2) $$c_{i}=\sum_{j=1}^{N}c_{i-1}h_{j}\;b_{i}=\sum_{j=1}^{N}c_{i-1}g_{j}$$

**Figure 1:**
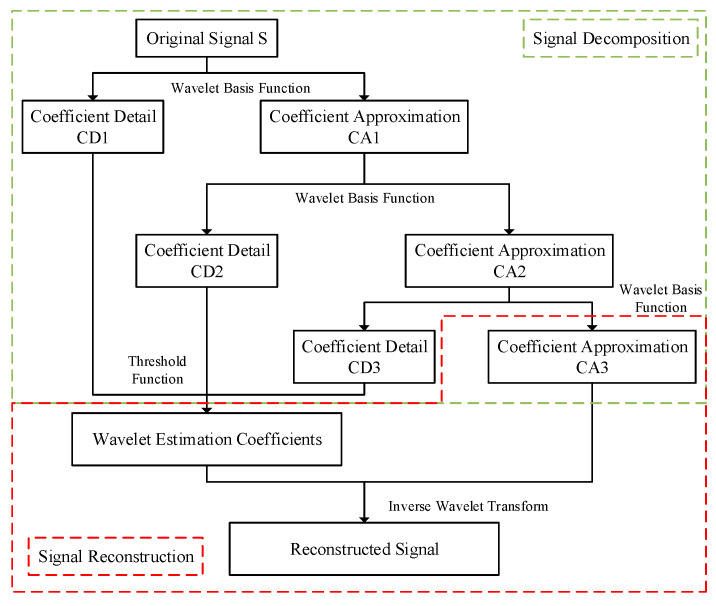
Schematic diagram of wavelet decomposition and reconstruction.

In the equation, N is sampling points; i is the number of decomposition layers; j is scale metric space of filters; $h_{j}$, $g_{j}$ are low-pass filters and high-pass filters, respectively; $c_{i}$, $b_{i}$ are low-frequency wavelet coefficients (the coefficient approximation, CAi), high-frequency wavelet coefficients (the coefficient detail, CDi) (Figure 1).

Threshold function denoising is a crucial step in the process of wavelet threshold denoising. Its principle is to select a certain threshold based on the noise level and signal characteristics, and perform zeroing or proportional reduction on wavelet coefficients of different sizes to achieve the purpose of filtering the noise signal. The process of wavelet reconstruction consists of recombining the wavelet estimated coefficients and approximate coefficients processed by a threshold function, and the mathematical expression for this process can be expressed as in Equation (3).

(3) $$c_{i-1}=\sum_{j=1}^{N}c_{i}h_{j}+\sum_{j=1}^{N}\omega_{i}g_{j}$$

The meaning represented by the parameters in the equation is the same as in Equation (2); $\omega_{i}$ is the wavelet coefficient value before the i-th layer processing.

#### 2.1.2. The Denoising Model of the BT-WTD

In wavelet threshold denoising, the threshold function reflects different processing methods for wavelet coefficients outside the threshold range. After the wavelet decomposition of acoustic signals, it is necessary to use the threshold function to process the approximation coefficients at different decomposition levels to obtain the wavelet estimation coefficients. Therefore, the design of the threshold function is at the core of the wavelet threshold denoising method.

In the threshold processing of signals, the problem of discontinuous points in the hard threshold function can cause additional oscillations in the reconstructed signal, and the smoothness of the processed signal will be greatly reduced compared to the original signal. When using a soft threshold function for signal processing, this processing method at $\left|\omega \right|\geq \lambda$ will result in a constant bias between $\omega_{\lambda }$ and λ, subsequently affecting the accuracy of the reconstructed signal. The comparison of the characteristics of using soft and hard threshold functions for wavelet denoising is shown in Table 1.

In order to address the shortcomings of the classical soft threshold and hard threshold functions, this paper proposes a biparameter and trisegment wavelet denoising threshold function with characteristics such as continuity, constant deviation, symmetry, and flexibility. The new threshold function with exponential functions and adjustment parameters divides the processing interval into three segments in the form of two thresholds: proportional reduction interval, transitional reduction interval, and zero setting interval. The expression for the new threshold function is as follows:

(4) $$\omega_{\lambda }=\left\{\begin{array}{cc} \mathrm{sgn}(\omega )\cdot (\left|\omega \right|-\frac{\lambda }{1+e^{\alpha \sqrt{\left(\left|\omega \right|-\lambda )(\left|\omega \right|-\lambda_{0}\right)}}}) & \left|\omega \right|>\lambda \\ \mathrm{sgn}(\omega )\cdot (\frac{\left|\omega \right|-\lambda_{0}}{\lambda -\lambda_{0}})\cdot \frac{\lambda }{1+e^{\alpha \sqrt{\left(\left|\omega \right|-\lambda )(\left|\omega \right|-\lambda_{0}\right)}}} & \lambda_{0}\leq \left|\omega \right|\leq \lambda \\ 0 & \left|\omega \right|<\lambda_{0} \end{array}\right.$$

In the formula, e is the natural constant, λ and $\lambda_{0}$ are both thresholds, satisfying $\lambda_{0}=\beta \lambda$. α and β are adjustable parameters, where $\alpha \in \left(0,+\infty \right)$ and $\beta \in \left(0,1\right)$. ω is the wavelet coefficient obtained through decomposition. The rationale for choosing this range of values will be provided in Section 2.2.3.

**Figure 2:**
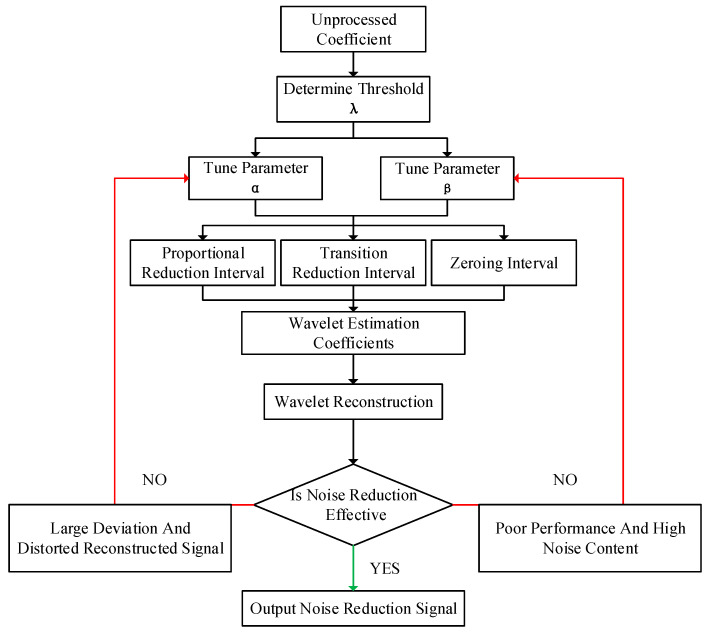
Denoising process of the BT-WTD algorithm.

This threshold function achieves the flexible adjustment of the threshold range and constant bias through two adjustable parameters. When the wavelet decomposition coefficient is larger than the threshold, it can quickly approximate the curve of the hard threshold function, reduce the constant bias, and avoid signal distortion (proportional reduction interval). When the wavelet decomposition coefficients are between two thresholds, this can avoid the occurrence of the pseudo-Gibbs problem (transition reduction interval). When the wavelet decomposition coefficient is less than the threshold, the wavelet coefficients are zeroed to preserve more useful signal features. This not only enhances the correlation between the wavelet coefficients in the soft threshold function but also compensates for the discontinuity at the threshold point in the hard threshold function. The flowchart of the BT-WTD algorithm for the acoustic signal denoising method proposed in this paper is shown in Figure 2.

### 2.2. Analysis of BT Threshold Function Characteristics

#### 2.2.1. Continuity

Due to the presence of two thresholds in the proposed threshold function, it is necessary to prove the continuity of the piecewise function separately for both threshold points.

At the threshold point λ, the following applies:

- When $\left|\omega \right|\to \;\lambda_{+}$,
  (5) $$f(\lambda_{+})=\underset{\left|\omega \right|\to \lambda_{+}}{lim}\mathrm{sgn}(\omega )\cdot (\left|\omega \right|-\frac{\lambda }{1+e^{\alpha \sqrt{\left(\left|\omega \right|-\lambda )(\left|\omega \right|-\lambda_{0}\right)}}})=\lambda -\frac{\lambda }{2}=\frac{\lambda }{2}$$
- When $|\omega |\to \lambda_{-}$,
  (6) $$f(\lambda_{-})=\underset{\left|\omega \right|\to \lambda_{-}}{lim}\mathrm{sgn}\;(\omega )\cdot (\frac{\left|\omega \right|-\lambda_{0}}{\lambda -\lambda_{0}})\cdot \frac{\lambda }{1+e^{\alpha \sqrt{\left(\left|\omega \right|-\lambda )(\left|\omega \right|-\lambda_{0}\right)}}}=\frac{\lambda }{2}$$

Thus, it can be concluded that $f(\lambda_{+})=f(\lambda_{-})$, so the improved threshold function is continuous at the threshold point λ.

At the threshold point $\lambda_{0}$, the following applies:

- When $\left|\omega \right|\to \;\lambda_{0+}$,
  (7) $$(\lambda_{0+})=\underset{\left|\omega \right|\to \lambda_{0+}}{lim}\mathrm{sgn}\;(\omega )(\frac{\left|\omega \right|-\lambda_{0}}{\lambda -\lambda_{0}})\frac{\lambda }{1+e^{\alpha \sqrt{\left(\left|\omega \right|-\lambda )(\left|\omega \right|-\lambda_{0}\right)}}}=0$$
- When $\left|\omega \right|\to \;\lambda_{0-}$,
  (8) $$f(\lambda_{0-})=0$$

Thus, it can be concluded that $f(\lambda_{0+})=f(\lambda_{0-})$, so the improved threshold function is continuous at the threshold point $\lambda_{0}$.

**Figure 3:**
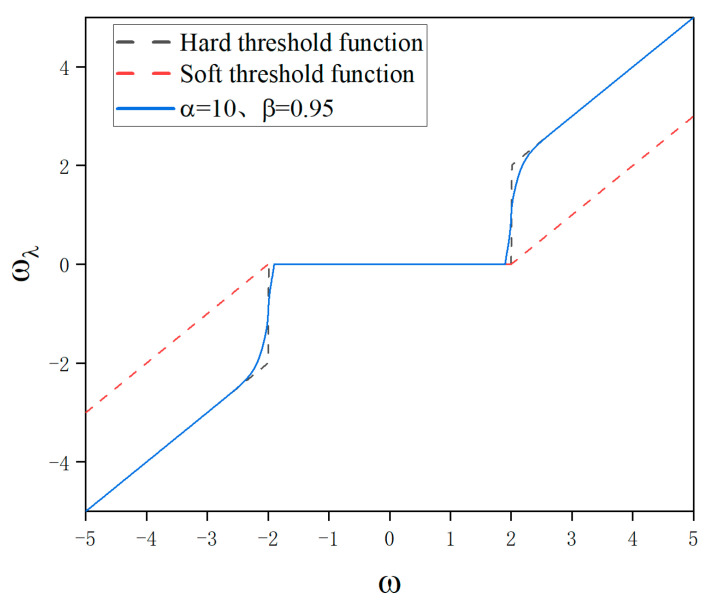
Comparison of the new threshold function and the original threshold function.

From the above proof process and continuity definition, it can be seen that the improved threshold function is both continuous at two threshold points λ and $\lambda_{0}$. Taking λ=2, it can be seen from the function graph shown in Figure 3 that the piecewise function is continuous within its domain.

#### 2.2.2. Parity

To verify the parity of the piecewise function f(w), it is only necessary to prove the mathematical equivalence between f(ω) and f(−ω). From the properties of the piecewise function and the values of its domain, the following can be inferred:

(9) $$\begin{array}{cc} s\mathrm{gn}(-\omega )\cdot (\left|\omega \right|-\frac{\lambda }{1+e^{\alpha \sqrt{\left(\left|\omega \right|-\lambda \right)\left(\left|\omega \right|-\lambda_{0}\right)}}})=-s\mathrm{gn}(\omega )\cdot (\left|\omega \right|-\frac{\lambda }{1+e^{\alpha \sqrt{\left(\left|\omega \right|-\lambda )(\left|\omega \right|-\lambda_{0}\right)}}}) & \left|\omega \right|>\lambda \\ \mathrm{sgn}\;(-\omega )\cdot (\frac{\left|\omega \right|-\lambda_{0}}{\lambda -\lambda_{0}})\cdot \frac{\lambda }{1+e^{\alpha \sqrt{\left(\left|\omega \right|-\lambda )(\left|\omega \right|-\lambda_{0}\right)}}}=-s\mathrm{gn}(\omega )\cdot (\frac{\left|\omega \right|-\lambda_{0}}{\lambda -\lambda_{0}})\cdot \frac{\lambda }{1+e^{\alpha \sqrt{\left(\left|\omega \right|-\lambda )(\left|\omega \right|-\lambda_{0}\right)}}} & \lambda_{0}\leq \left|\omega \right|\leq \lambda \\ 0=0 & \left|\omega \right|<\lambda_{0} \end{array}$$

Therefore, within the corresponding domain range, f(−ω)=−f(ω) holds true. From Figure 3, it can also be observed that this piecewise function is symmetric at about ω = 0 within its domain and has f(ω) = 0. Thus, it can be concluded that this new threshold function is an odd function.

#### 2.2.3. Bias

The ultimate goal of wavelet decomposition is to minimize the value of $\left|\omega_{\lambda }-\omega \right|$; when evaluating the denoising performance of the threshold function, it is necessary to compute the deviation between the improved threshold function and the hard threshold function outside the threshold range. Due to the fact that both the hard threshold function and the improved threshold function are odd functions within the domain range, when calculating the difference between both, only the change in the difference over the range ω≥λ needs to be calculated.

- When ω→+∞,
  (10) $$\underset{\omega \to +\infty }{lim}(\left|\omega_{\lambda }\right|-\omega )=\underset{\omega \to +\infty }{lim}(\frac{\lambda }{1+e^{\alpha \sqrt{\left(\left|\omega \right|-\lambda )(\left|\omega \right|-\lambda_{0}\right)}}})=\underset{\omega \to +\infty }{lim}(\frac{\lambda }{1+e^{\alpha \omega }})$$

**Figure 4:**
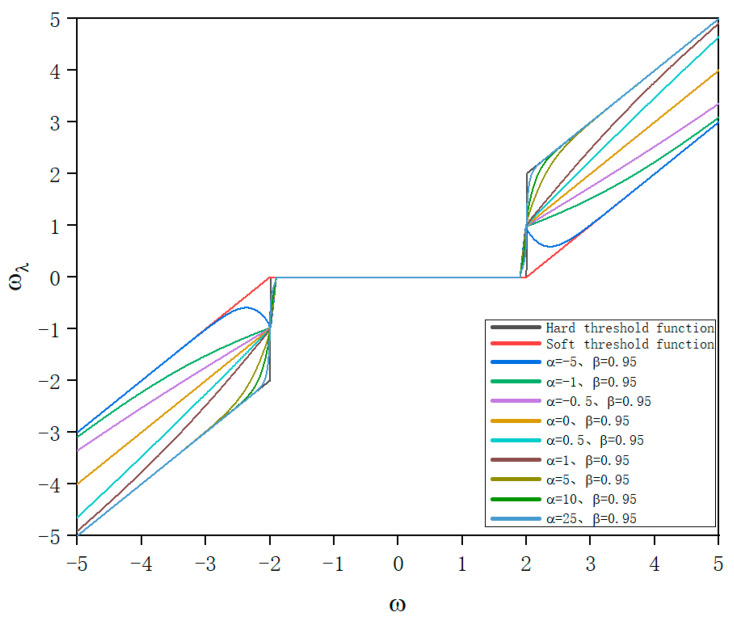
Threshold functions under different values of adjustment parameter α.

From the definition of the exponential function, we know that when α>0, $\underset{\omega \to +\infty }{lim}(\left|\omega_{\lambda }\right|-\omega )=0$; when α=0, $\underset{\omega \to +\infty }{lim}(\left|\omega_{\lambda }\right|-\omega )=\frac{\lambda }{2}$; when α<0, $\underset{\omega \to +\infty }{lim}(\left|\omega_{\lambda }\right|-\omega )=\lambda$. From Figure 4, the characteristics of the function graphs with different values of parameter α under the condition of ω→+∞ and β=0.95 can also be observed. Therefore, in order to reduce the deviation of the improved threshold function when the wavelet decomposition coefficients tend to infinity, the range of values for the adjustable parameter α should be α∈(0,+∞), and in verification of the relevant properties of the improved threshold function, α only considers the case of taking positive numbers.

#### 2.2.4. Asymptote

From the above proof process, it can be seen that when α∈(0,+∞) and $\left|\omega \right|\geq \lambda$, we have

(11) $$\underset{\omega \to \infty }{lim}\frac{\omega_{\lambda }}{\omega }=\underset{\omega \to \infty }{lim}\frac{\omega -\frac{\lambda }{1+e^{\alpha \sqrt{\left(\omega -\lambda )(\omega -\lambda_{0}\right)}}}}{\omega }=\underset{\omega \to \infty }{lim}(1-\frac{\lambda }{\omega (1+e^{\alpha \sqrt{\left(\omega -\lambda )(\omega -\lambda_{0}\right)}})})=1$$

From the above equation, it can be seen that when $\left|\omega \right|\geq \lambda$, the asymptote of the improved threshold function is $f\left(\omega \right)=\omega$, and the slope of the asymptote of the hard threshold function is consistent with the improved threshold function.

#### 2.2.5. Biparameter Analysis

In the new threshold function, these two adjustable parameters introduced are the slope adjustment parameter α and the threshold range adjustment parameter β. From Figure 4, it can be observed that when the threshold range adjustment β is set to 0.95, as the range of values for α increases from 0 to positive infinity, the asymptote of the new threshold function approaches y=x, which is the same as the asymptote of the hard threshold function. This significantly reduces the deviation of wavelet coefficients outside the threshold range in the wavelet denoising process.

**Figure 5:**
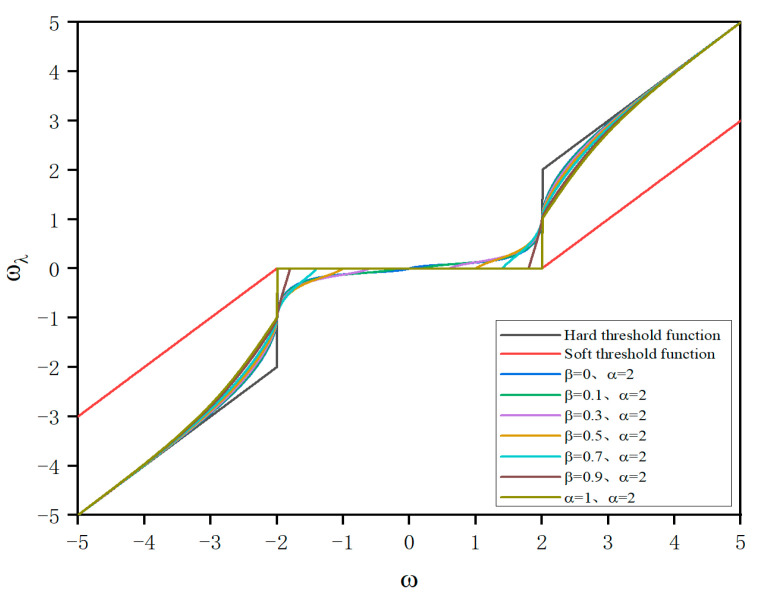
Threshold functions under different values of adjustment parameter β.

From Figure 5, it can be concluded that when α=2 is fixed and the threshold range adjustment parameter β changes from 0 to 1, the zeroing interval of the threshold function increases from small to large. The new threshold function exhibits varying capabilities in handling wavelet coefficients within a given threshold range from strong to weak. Therefore, by adjusting the range of β, it is possible to flexibly handle noise signals across various frequency bands. This approach can effectively preserve the useful features of the original signal during denoising while achieving a significant reduction in noise signal.

### 2.3. Denoising Quantitative Evaluation Indicators

The signal-to-noise ratio (SNR), Root Mean Square Error (RMSE), and Normalized Correlation Coefficient (NCC) are commonly used evaluation indicators to assess the quality of denoising effects. This paper will quantitatively evaluate the denoising model BT-WTD through these three indicators.

The mathematical expression for the SNR is given in Equation (12).

(12) $$SNR=10\mathrm{lg}\frac{\sum_{i=1}^{n}{x_{\left(i\right)}}^{2}}{\sum_{i=1}^{n}(x_{\left(i\right)}-{x_{\left(i\right)}}^{\prime }{)}^{2}}$$

In the equation, $x_{\left(i\right)}$ represents the denoised signal sequence, ${x_{\left(i\right)}}^{\prime }$ represents the original (without noise) sequence, and *n* is the length of the signal sequence.

The mathematical expression for the RMSE is given in Equation (13).

(13) $$RMSE=\sqrt{\frac{\sum_{i=1}^{n}(x_{\left(i\right)}-{x_{\left(i\right)}}^{\prime }{)}^{2}}{n}}$$

The mathematical expression for the NCC is given in Equation (14).

(14) $$NCC=\frac{\sum_{i=1}^{n}As_{\left(i\right)}Ad_{\left(i\right)}}{\sqrt{\sum_{i=1}^{n}A{s^{2}}_{\left(i\right)}\sum_{i=1}^{n}A{d^{2}}_{\left(i\right)}}}$$

In the equation, As refers to the original signal (i.e., the clean signal), and Ad refers to the signal sequence after denoising.

## 3. Simulation Experiment Verification

### 3.1. Signal Simulation

The vibration and acoustic signals of faulty conveyor belt rollers usually contain modulated components of stationary signals and periodic impacts. In order to verify the denoising performance of the BT algorithm on one-dimensional data signals, referring to the vibration and acoustic signal models of roller bearings, the simulation of stationary signals is achieved using the characteristics of sine signals. Based on the periodic impact characteristics generated during local faults in the outer ring of the bearings, the simulation of fault impact signals was carried out.

#### 3.1.1. Sine Wave Simulation Signal

Sine function:

(15) $$x_{1}(t)=\sin(2\pi \cdot f_{n}\cdot t)$$

In the formula, function $x_{1}(t)$ is the expression of the sine signal function, $f_{n}$ is the frequency of the simulated sine signal, and t is the sampling time. The parameters include the sampling frequency fs=5120 Hz, number of samples N=5120, sine signal frequency $f_{n}=15\;Hz$, and sampling time t=1 s.

#### 3.1.2. Periodic Impact Simulation Signal

In the process of constructing the periodic impact signal, it is assumed that the outer ring of the bearing is stationary and there is a localized fault, while the inner ring rotates at a certain speed along with the shaft. The periodic impacts caused by the fault can be represented as a sine function with amplitude decaying exponentially.

(16) $$x_{2}(t)=y_{0}e^{-2\pi f_{n}gt}\sin(2\pi f_{n}t\sqrt{1-g^{2}})$$

In the formula, $y_{0}$ is the amplitude displacement, g is the damping coefficient of the bearing system, and $f_{n}$ is the natural frequency of the bearing system.

Based on the above expression of the impact signal, the amplitude displacement $y_{0}=5\;m\cdot s^{-2}$, the natural frequency of the bearing system $f_{n}=3000\;Hz$, the damping coefficient g=0.04, the sampling frequency fs=20 kHz, and the number of samples N=20,000 are determined.

#### 3.1.3. Adding Gaussian White Noise to Signal

In the real environment, noise is not only caused by a single source. It is usually a superposition of vibration or acoustic signals from multiple factors and sources. When simulating noise addition to simulated signals, Gaussian white noise is commonly used to approximate this complex and uncertain noise distribution situation. Its expression can be represented as

(17) $$x_{noise(t)}=N\sqrt{P_{noise}}=N\sqrt{\frac{P_{signal}}{10^{\frac{SNR}{10}}}}=N\sqrt{\frac{\sum_{i}^{N}\frac{{x_{i}}^{2}}{N}}{10^{\frac{SNR}{10}}}}$$

In the formula, *N* is the length of the original signal, $P_{noise}$ and $P_{signal}$ are the average power of the Gaussian white noise signal and the original signal, respectively, and the SNR is the signal-to-noise ratio of the added Gaussian white noise.

In order to fully validate the effectiveness and robustness of the BT-WTD algorithm denoising model, six different noise signals with signal-to-noise ratios of −15 dB, −10 dB, −5 dB, 5 dB, 10 dB, and 15 dB will be added to the stationary simulation signal and periodic impact simulation signal.

### 3.2. Selection of Denoising Model Basic Parameters

Selection of threshold: It can be understood from the characteristics of the noise distribution that under the first decomposition scale, the wavelet decomposition coefficients have the highest noise content, which rapidly decays as the wavelet threshold decomposition levels increase. Considering the correction of noise variance on the threshold size, the fixed threshold is adjusted according to the formula as shown in Equation (18). Finally, the calculation formula for the fixed threshold is shown in Equation (19).

(18) $$\sigma_{i}=\frac{median(\left|\omega_{i}\right|)}{0.6745}$$

(19) $$\lambda =\sigma_{i}\sqrt{2\mathrm{lg}(N)}$$

In the formula, median() is the median function, capable of calculating the median of the wavelet decomposition coefficients under a given decomposition scale. i represents the number of levels of the decomposition scale. Since there are more noise signal components in the first decomposition scale, i=1 is used.

Selection of wavelet basis functions: The acoustic and vibration signals of faulty rollers in belt conveyors are both impact signals. When denoising the signals, wavelet basis functions with shapes closest to the faulty impact signals can best preserve the useful signal characteristics and improve the signal-to-noise ratio. Therefore, this model chooses Coif 10 as the wavelet basis function for the denoising preprocessing of acoustic and vibration signals.

Selection of decomposition level: In the process of performing wavelet threshold decomposition on the signal, more decomposition levels will make the distinction between the noise signal and useful features more noticeable, which is more conducive to separating the two signals. However, more decomposition levels will also result in the greater distortion of the reconstructed signal after wavelet denoising. Therefore, taking into account the characteristics of the roller acoustic signal and the sampling frequency, the wavelet decomposition level is set to five layers.

### 3.3. Simulation Signal Denoising Verification

After selecting Coif 10 as the wavelet base function and setting the decomposition level to 5, the performance of the BT-WTD algorithm will be verified. The hard thresholding function, soft thresholding function, and BT-WTD algorithm will be used to denoise two types of simulation signals. The quantitative evaluation of the denoising results will be carried out using three indicators: the waveform similarity, signal-to-noise ratio, and Root Mean Square Error.

**Figure 6:**
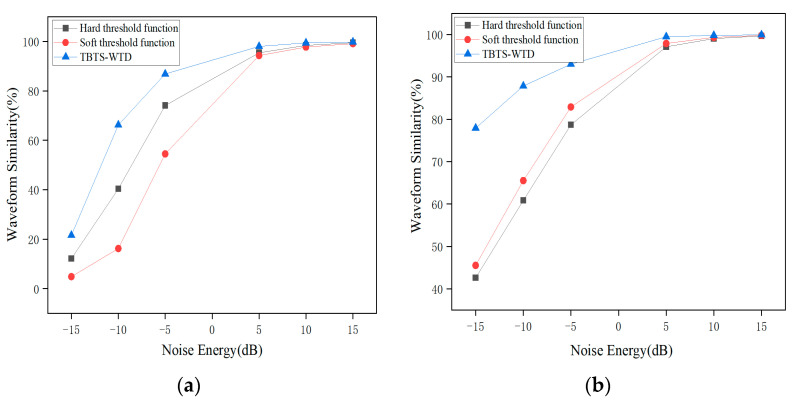
Waveform similarity under different wavelet threshold functions: (**a**) impulse signal; (**b**) stationary signal.

As shown in Figure 6, after denoising the impulse signals with Gaussian white noise of different signal-to-noise ratios, the denoising method using the BT-WTD algorithm significantly outperforms the other two methods in terms of the waveform similarity. The denoising performance for the impulse signals is improved by 308.01% using the BT-WTD algorithm.

**Figure 7:**
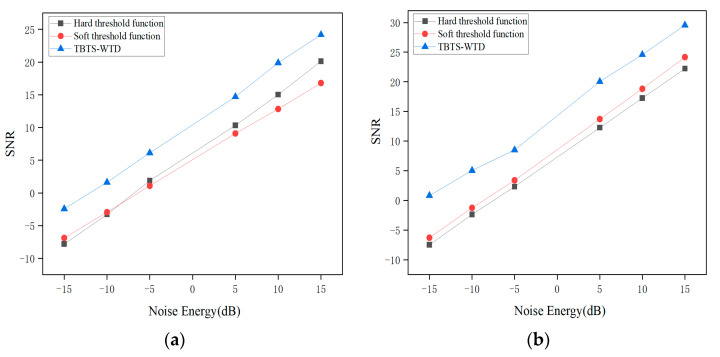
Signal-to-noise ratio under different wavelet threshold functions: (**a**) impulse signal, (**b**) stationary signal.

As shown in Figure 7, after denoising the impulse signals with Gaussian white noise of different signal-to-noise ratios, the BT-WTD algorithm also outperforms the other two methods in terms of the signal-to-noise ratio. The denoising performance of the impact signal is maximally improved by 32.36%, while the denoising performance of the stationary signal is maximally improved by 267.97%.

**Figure 8:**
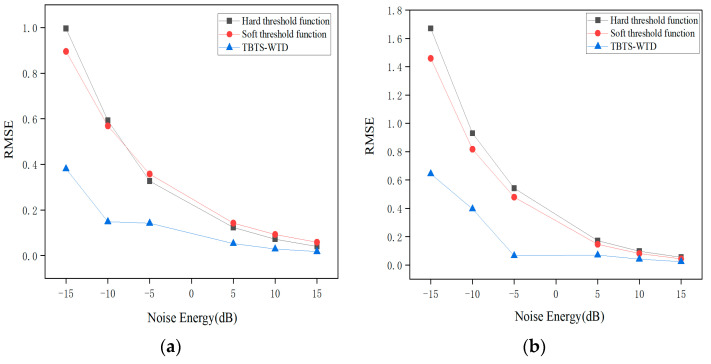
Root Mean Square Error under different threshold functions: (**a**) impulse signal, (**b**) stationary signal.

As shown in Figure 8, after denoising the impulse signals with Gaussian white noise of different signal-to-noise ratios, the Root Mean Square Error of the BT-WTD algorithm is significantly lower than the other two methods. The maximum optimization of the Root Mean Square Error for the impact signal is 61.796%. And for the stationary signal, the maximum optimization is 61.480%.

**Figure 9:**
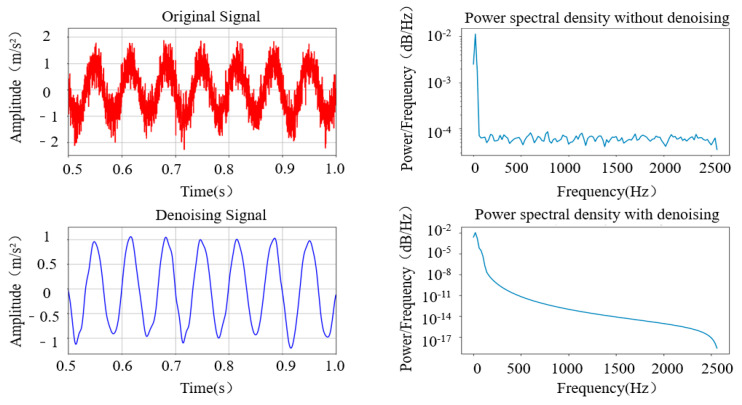
Comparison of characteristics of simulated sine signal before and after denoising.

**Figure 10:**
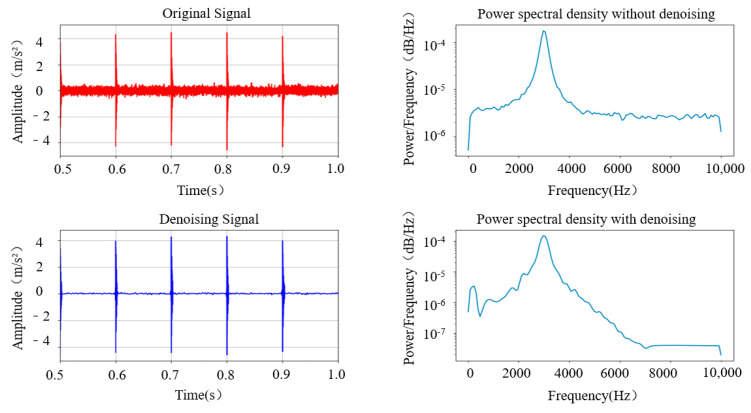
Comparison of characteristics of impact signal before and after denoising.

The experimental results above indicate that using the BT-WTD denoising model can effectively denoise stationary and impact simulation signals. The performance of the BT-WTD algorithm is improved significantly compared to the hard threshold functions and soft threshold functions. As shown in Figure 9, during the denoising process of the stationary signal, a comparison is made between the signal characteristics before and after denoising in the environment with Gaussian white noise added at a signal-to-noise ratio of 5 dB. In terms of the time domain, the BT-WTD denoising method can effectively denoise the simulated signal while ensuring that the signal does not exhibit distortion as much as possible. In terms of the frequency domain, the frequency domain characteristics of the signals before and after denoising are analyzed using power spectral density. The energy level of the signal before denoising is between 1 × 10^−2^ and 1 × 10^−5^, while the frequency energy level after denoising is between 1 × 10^−2^ and 1 × 10^−18^. The denoising effect is particularly noticeable at both low and high frequencies. The denoising algorithm can significantly reduce the noise energy of the simulation signal at the overall characteristic frequency and preserve the energy of the sine signal well at 15 Hz. As shown in Figure 10, during the denoising process of the impact signal, Gaussian white noise with the same 5 dB signal-to-noise ratio is added. In terms of the time domain, the BT-WTD denoising model can effectively preserve the impact characteristics of the signal and reduce non-impact signal time-domain indicators. In terms of the frequency domain, the energy of the signal at the natural frequency of 3000 Hz remains unchanged before and after denoising while the overall frequency energy distribution of the signal changes from [1 × 10^−3^, 1 × 10^−7^] to [1 × 10^−3^, 1 × 10^−8^] after denoising, with a significant denoising effect at high frequencies.

## 4. Experimental Data Collection and Validation

### 4.1. Experimental Data Collection

**Figure 11:**
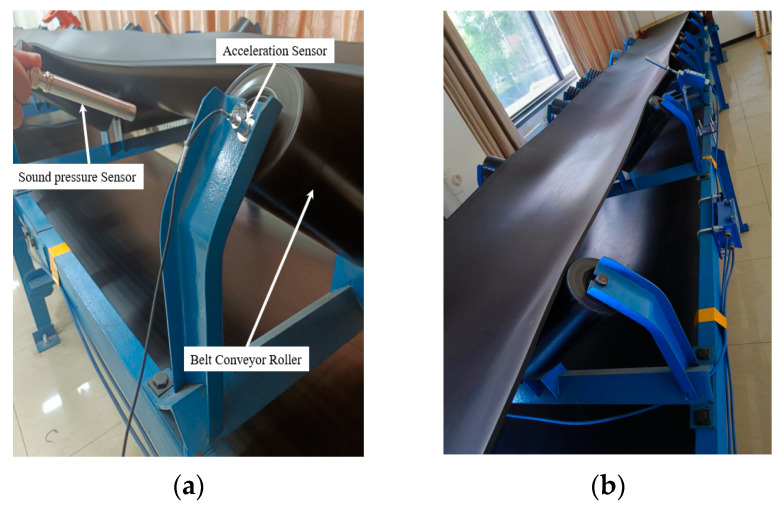
Layout of experimental platform and sensors: (**a**) sensor layout, (**b**) belt conveyor idler experiment platform.

**Figure 12:**
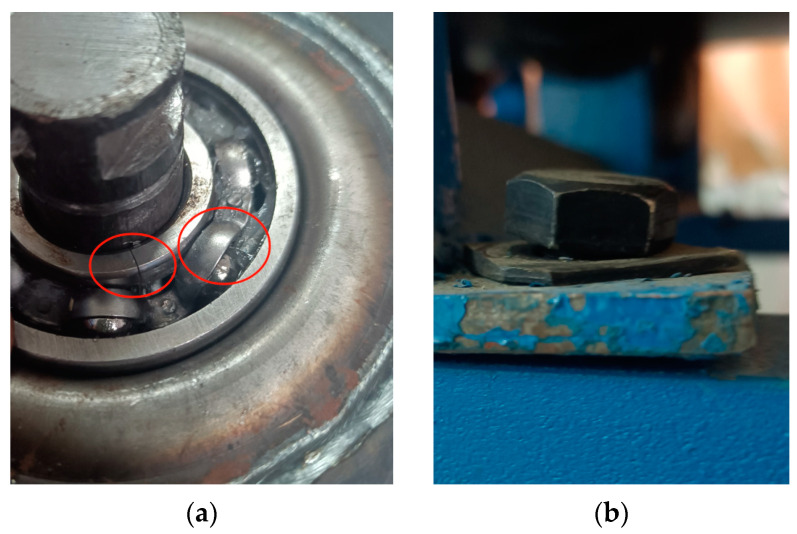
Simulated working conditions of roller bearing failure and abnormal support frame: (**a**) bearing failure (The red circle from left to right indicates that the inner ring of the bearing is broken and the cage is deviated), (**b**) abnormal support frame for roller support.

Due to the high-speed, long-term, and high-load working characteristics of the roller, problems such as coal accumulation, high temperature of bearings, and vibration between equipment can cause problems such as roller jamming, bearing failure, and support frame failure, ultimately leading to roller failure. Therefore, this paper completes the collection of experimental data by simulating the following four working conditions of the roller: maintain consistency in the model and size of the rollers, and fix them with ropes to simulate the conditions of a roller jam fault; simulate bearing failure by damaging the inner race and retainer of the bearing; and simulate the loosening fault condition of the support frame by loosening the fixing bolts of the roller support frame. And then, we collected acoustic and vibration signals under normal working conditions and three types of fault conditions, with a sampling frequency of 12.8 kHz. The parameters of the experimental platform equipment and the acquisition instrument are shown in Table 2. The layout of the experimental platform and sensors is shown in Figure 11. The simulated working conditions to the bearing’s failure and the loosening of the fixing bolts on the support bracket are shown in Figure 12.

### 4.2. Denoising Analysis of Experimental Data

**Figure 13:**
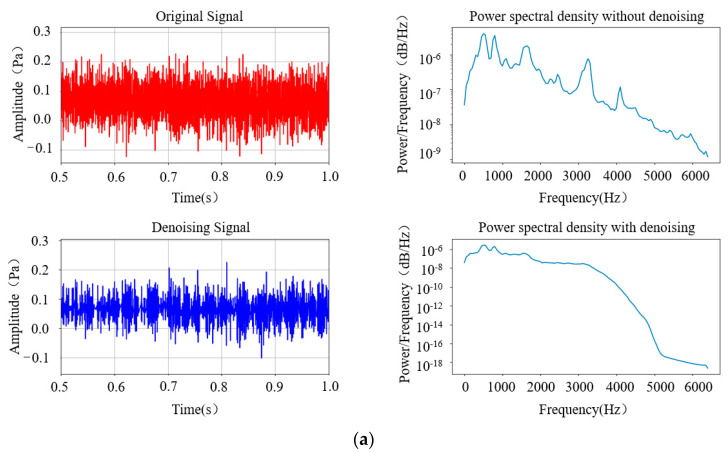

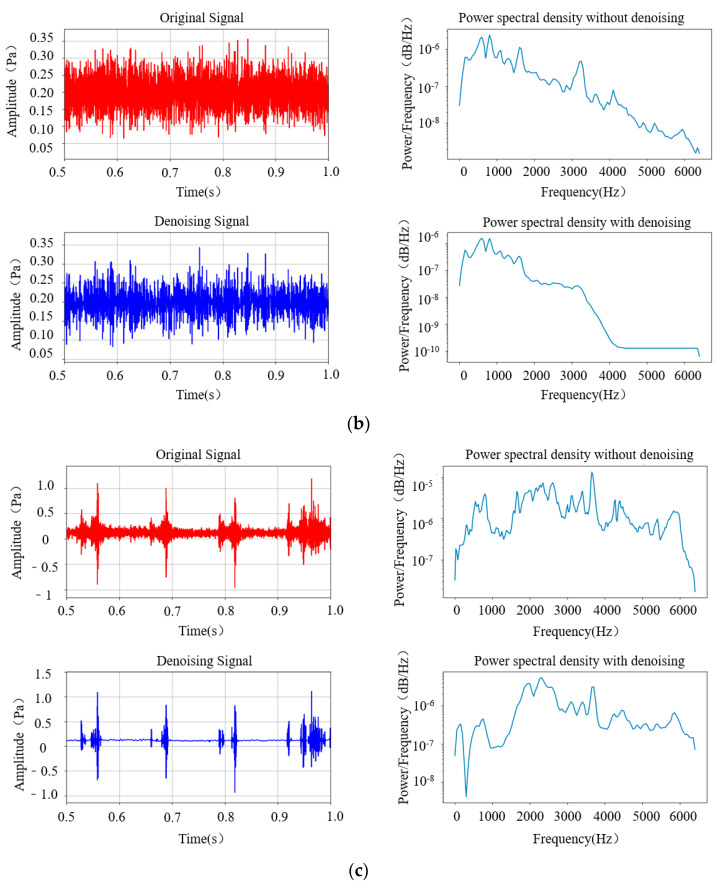

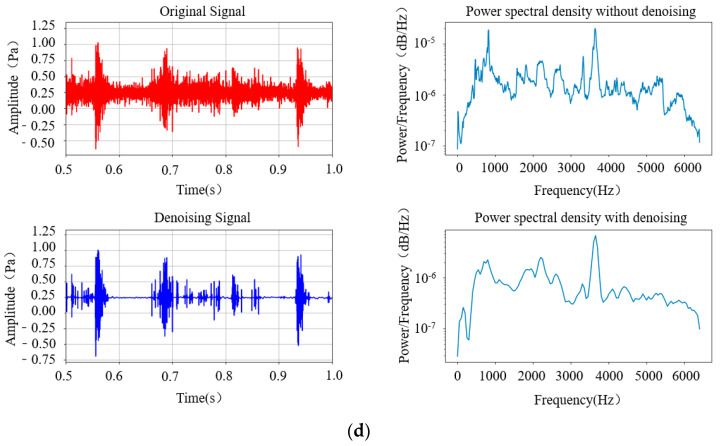
Acoustic signals of idler rollers in different operating conditions: (**a**) normal operating condition, (**b**) roller jamming, (**c**) bearing failure, (**d**) support frame abnormality.

The collected signals are input into the denoising model based on the BT-WTD algorithm. As shown in Figure 13, it can be seen that the denoising model proposed in this paper has a significant denoising effect on the acoustic signals under four different working conditions. From the time domain perspective, the time domain indicators in the original signal are retained, and to some extent, the noise components in the signal are filtered out, and the overall signal does not have any distortion problems in the reconstructed signal; from the frequency domain perspective, by comparing the power spectral density maps of the signal before and after denoising, it can be seen that the energy of the denoised signal has decreased in the overall frequency range, and the main frequency energy distribution under various operating conditions has been well preserved. It can be considered that, after denoising, the proportion of noisy energy in the original acoustic signal of the roller has been reduced, and the influence of noise interference in the acoustic signal collected against a strong noise background has been weakened.

**Figure 14:**
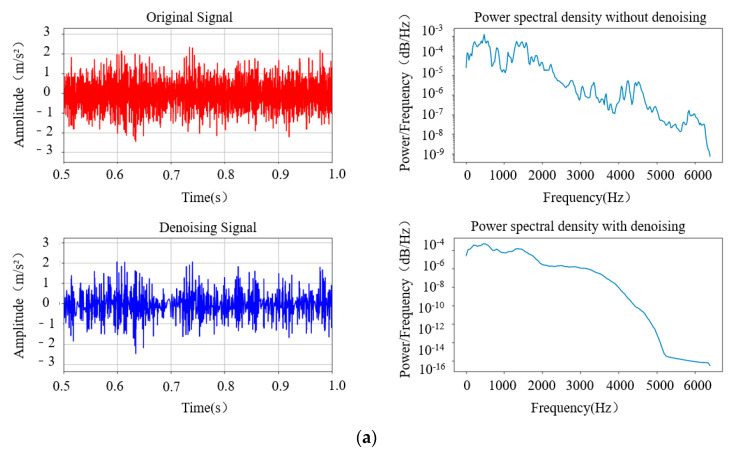

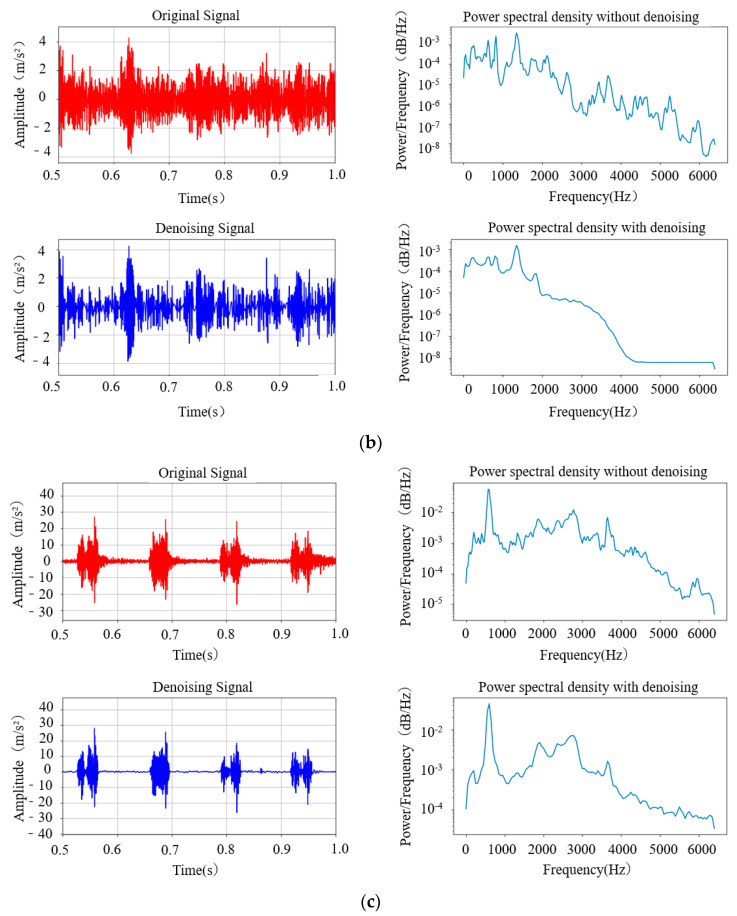

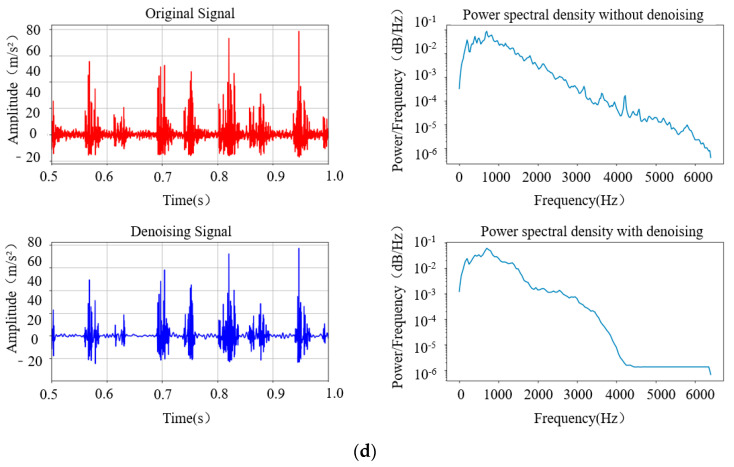
Vibration signals of idler rollers in different operating conditions: (**a**) normal operating condition, (**b**) roller jamming, (**c**) bearing failure, (**d**) support frame abnormality.

To validate the generalization ability of the denoising model, the collected vibration signals were also pre-processed using the denoising model. As shown in Figure 14, it can be seen that before and after denoising, the denoising effect of the proposed denoising model is still significant and able to simultaneously preprocess both acoustic and vibration signals with strong denoising capabilities. This denoising model has been proven to exhibit good denoising effects in both the low- and high-frequency bands of the entire signal, while also reducing the impact of noise energy in the frequency domain of the signal.

## 5. Conclusions

In this paper, a denoising model based on the BT-WTD algorithm was built and the superiority of the threshold function used in the model was theoretically discussed. By using two adjustment parameters, the interval length of the denoising threshold and the asymptote slope of the threshold function outside the threshold interval can be flexibly adjusted. And this method has been proven to improve the generalization ability of the denoising model. The reliability of the denoising model based on the BT-WTD algorithm was verified through denoising experiments of simulated signals and roller signals, which compensated for the shortcomings of the soft threshold functions and hard threshold functions. This effectively solved the problem of signal distortion after denoising in the preprocessing process of the acoustic and vibration signals of belt conveyor rollers. This method has ultimately been proven to retain the original advantages of wavelet threshold denoising methods and improve the robustness and generalization ability of denoising models.

## Figures and Tables

**Table 1:** Characteristics of soft and hard threshold functions.

Characteristics	Hard Threshold Function	Soft Threshold Function
RMSE	Low	High
Smoothness	Bad	Good
Continuity	Good	Bad
Generation of additional oscillations	Yes	No

**Table 2:** Parameters of each device in the experimental platform.

Equipment Name	Equipment Parameters
Three-phase asynchronous motor	Model YE2VP132M-4, rated speed 1455 r/min
Grooved buffer roller	Inner diameter × outer diameter × roller length: 45 mm × 133 mm × 380 mm
Signal data acquisition instrument	INV3018CT
Accelerometer	ICP INV9822
Acoustic pressure sensor	ICP INV9206

## Data Availability

No new data were created or analyzed in this study. Data sharing is not applicable to this article.

## Abbreviations

The following abbreviations are used in this manuscript:

BT	the function of biparameter and trisegment
SNR	signal-to-noise ratio
MSE	Mean Square Error
RMSE	Root Mean Square Error
NCC	Normalized Correlation Coefficient
